# Sarcomatoid Carcinoma of the Prostate

**DOI:** 10.1155/2013/631809

**Published:** 2013-04-04

**Authors:** Onur Açıkgöz, Eymen Gazel, Neslihan İnci Zengin, Yusuf Kasap, Ahmet Çamtosun, Ahmet Hamdi Yazıcıoğlu

**Affiliations:** ^1^Department of Urology, Turkey Yüksek İhtisas Training and Research Hospital, Ankara, Turkey; ^2^Department of Pathology, Turkey Yüksek İhtisas Training and Research Hospital, Ankara, Turkey

## Abstract

Sarcomatoid carcinoma of the prostate is among the rarest malignant neoplasm types and has been well known for its aggressive clinical course. Patient was admitted with the symptoms of lower urinary tract. Transurethral resection of prostate (TUR-P) was carried out. Revealing Gleason 5 + 3 = 8 prostate adenocarcinoma in TUR-P material. Thereby, a Radical Prostatectomy procedure was planned. In operation, frozen examination revealed adenocarcinoma metastasis to the obturator lymph node. The operation was terminated. In the postoperative 3rd month, the patient was re-admitted with acute urinary system symptoms. A cystoscopy performed and complete resection of the mass was performed. The pathological examination reported that the tumor was compatible with undifferentiated adenocarcinoma owing to presence of poorly differentiated tumoral cells and detection of adenocarcinoma in a relatively small (<1%) focus. 4 month after the operation, the patient underwent another cyctoscopic examination which revealed the prostatic lounge and most of the bladder lumen to be filled with tumoral tissue. The tumoral tissues was resected incompletely. This material was diagnosed to be “Sarcomatoid Malignant Tumor” upon the new evidences of progressive dedifferentiation and predominant sarcomatoid appearance, compared with the former TUR-P materials. Subsequent PET-CT scan depicted multiple metastasis. The patient was referred to oncology department. In conclusion, sarcomatoid carcinoma is a malignant variant that brings along diagnostic and treatment difficulties.

## 1. Introduction

Sarcomatoid carcinoma of the prostate is among the rarest malignant neoplasm types and has been well known for its aggressive clinical course. To date, there have been around 100 cases reported in the literature [1]. The most recent World Health Organization (WHO) classification does not distinguish clinically between carcinosarcoma and sarcomatoid carcinoma as two distinct lesions, although there appears to be cases characterized, regarding pathological diagnoses, with differentiation of prostatic adenocarcinoma into sarcomatoid carcinoma and some others characterized with primary carcinosarcoma containing both the epithelial and the mesenchymal cells. The term “sarcomatoid carcinoma” is used for both of these lesions [2]. It pursues an insidious course and generally becomes manifesting with symptoms related to bladder filling (nocturia, urinary urgency, etc.) and/or symptoms related to urination (dysuria, slow urine stream, hesitancy, terminal dribbling, feeling of residual urine, etc.) in adult and elderly populations. Rarely might the suffering cases be admitted with hematuria, perineal/rectal pain, or painful ejaculation [3].

Hereby, we reported the case of a 54-year-old male with histopathological findings compatible with differentiation of the prostate adenocarcinoma into the sarcomatoid carcinoma and intended to discuss the treatment protocol.

## 2. Case

**Figure 1:**
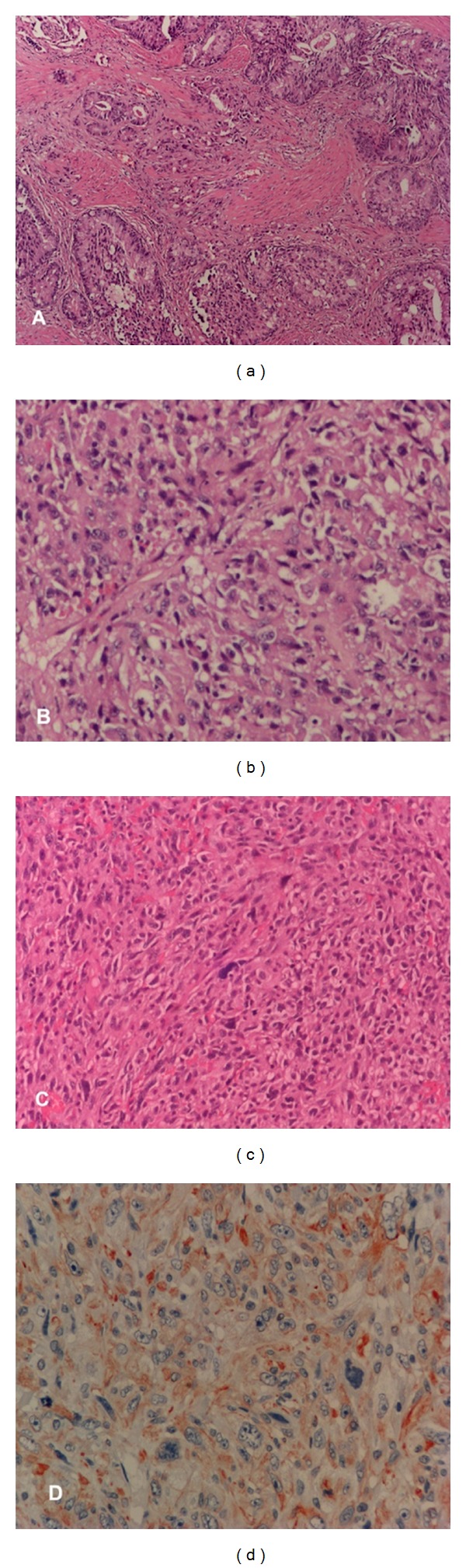
(a) Gleason 5 + 3 = 8 prostate adenocarcinoma in pathological examination of the first TUR-P material (an area which has more glands). (b) Undifferentiated tumor cells in pathological examination of the second TUR-P material. (c) Sarcomatous areas in the last TUR-P material (spindle and rare bizarre cells). (d) Sarcomatoid cells in immunohistochemical examination (vimentin positive/AEC chromagen).

A 54-year-old male was admitted to our clinic with the symptoms of lower urinary tract. His previous history revealed no family history regarding urinary cancer, being on an alpha-blocker therapy for the last 1 year, and nocturia (3-4 times a night), pollakiuria, and decrease in the urinary projection caliber of approximately 6-month duration. In the digital rectal examination, the prostate was palpated to possess grade 1 dimensions, benign surface, and soft consistency. Transrectal ultrasonography depicted a prostate gland of 40 cc volume with no trace regarding any ultrasonographic pathology. In the laboratory analysis, renal function tests, complete urinalysis, and ALP value were detected to be normal; PSA was measured to be 20.5 ng/mL. On the basis of these findings, the patient was advised to undergo an USG-guided transrectal biopsy and 16-quadrant biopsy procedure was implemented. Following the biopsy report indicating benign biopsy specimens, a transurethral resection of prostate was (TUR-P) carried out, revealing Gleason 5 + 3 = 8 prostate adenocarcinoma in the pathological examination of the TUR-P material (Figure 1(a)). Thereby, a radical prostatectomy procedure was planned for the patient. A constellation of imaging procedures, performed preoperatively, including abdominopelvic and thoracic CT scans and total body bone scintigraph, reported no pathological finding. Upon dissection of the patient for the operation, obturator lymph nodes were sampled for the frozen section examination, which then revealed adenocarcinoma metastasis to the right obturator lymph node. At this point, the operation was terminated without prostatectomy. In the postoperative period, the patient was placed on maximal androgen blockage therapy and then discharged. During the first follow-up visit at the end of the 1st postoperative month, the medical therapy was sustained due to the PSA value lower than 0.06 ng/mL. In the postoperative 3rd month, the patient was readmitted to our outpatient polyclinic with acute urinary system symptoms. An USG examination carried out at that time depicted hydronephrosis in the left kidney and a mass lesion of 4 cm in the longer axis in the prostatic lounge. A cystoscopy performed on the basis of these findings showed a mass lesion in the prostatic lounge which extended into the urinary bladder. A complete resection of the mass was performed. The pathological examination reported that the tumor was compatible with undifferentiated adenocarcinoma owing to the presence of poorly differentiated tumoral cells and detection of adenocarcinoma in a relatively small (<1%) focus (Figure 1(b)). Moreover, the pathology report further indicated that the tumor did not display staining for pancytokeratin, however, that undifferentiated tumors might be characterized with pancytokeratin negativity. The patient was sustained on medical therapy under close followup. 4 months after the operation, the patient presented to emergency with the complaint of inability to urinate. The laboratory analysis showed that PSA was 0.177 ng/dL, ALP was normal, BUN was 52 mg/dL, and creatinine level was 2.31 mg/dL. The patient underwent another cystoscopic examination which revealed the prostatic lounge and most of the bladder lumen to be filled with tumoral tissue. The tumoral tissues were resected incompletely and then sent for the pathological examination. This tumoral material was diagnosed to be “sarcomatoid malignant tumor” upon the new evidence of progressive dedifferentiation and predominant sarcomatoid appearance, compared with the former TUR-P materials (Figures 1(c) and 1(d)). Histopathological findings are summarized in Table 1.

Subsequent PET-CT scan depicted multiple metastasis in the lungs, liver, and thoracolumbar vertebrae. The patient was referred to the oncology department for the planning of radio- and chemotherapies, and he is still being followed up alive in the 9th postoperative month.

## 3. Discussion

Sarcomatoid carcinoma is one of the rare neoplasms of the prostate and pursues an insidious and aggressive clinical course. İn about half of all cases, the initial diagnosis was a usual acinar adenocarcinoma, followed by hormonal and/or radiation therapy, with a subsequent diagnosis of sarcomatoid carcinoma. The time between the initial diagnosis of acinar adenocarcinoma and the diagnosis of sarcomatoid carcinoma varied from 0.5 to 16 years, with a mean of 7 years. In our case this period was 8 months [4]. It is likely to grow locally and to make distant metastasis in a quite short period of time [1]. Should it be late for the surgery, the results stay far away from being satisfactory in such a neoplasm which is highly resistant to both chemo- and radiotherapies. The risk of one-year mortality was reported in various studies to be more than 20% [5]. Contrary to adenocarcinoma of the prostate, the most commonly seen type, the issue of inability of the sarcomatoid variant to raise PSA levels, results in diagnostic delays and admission of the suffering patients with late-stage symptoms. As in our case, involvement of the lower and upper urinary systems is possible owing to impingement of rapidly growing tumoral mass. In our case, differentiation of the adenocarcinoma to the sarcomatoid carcinoma is present; moreover, the patient was followed up under maximal androgen blocking therapy to keep PSA < 0.2 ng/mL during the disease period. However, the efficacy of hormone therapy observed on adenocarcinoma cannot be experienced on sarcomatoid variant, thus causing the presence of sarcomatoid component to be missed out in patients who are followed up by successive PSA measurements under hormone therapy. The definitive diagnosis of the disease can be established through pathological examination. Differential diagnosis includes some benign lesions (postoperative spindle cell nodule, etc.) or primary prostatic sarcoma, such as malignant phyllodes tumor, leiomyosarcoma, and malignant solitary fibrous tumor [6].

In conclusion, sarcomatoid carcinoma is a malignant variant that brings along diagnostic and treatment difficulties. Lack of experience in this subject hinders standardization of treatment modalities. Surgical resection of the curative tumors, adjuvant chemotherapy, and/or radiotherapy, together with palliative attempts in advanced-staged patients, constitute the general approach to this disease [5].

## Figures and Tables

**Table 1:** Histopathological findings of sarcomatoid tumor in our patient.

Light microscopy	High cellularity, spindle and pleomorphic cells with bizarre appearance, atypical mitotic figures, and necrosis.	
Immunohistochemical findings	Cytokeratin (AE1/AE3)	Negative
	Vimentin (V9)	Positive
	SMA	Positive
	Factor VIII	Negative
	C-kit (CD117)	Negative (rare, weak, granular positivity in the Golgi area)
	CD34	Negative

## References

[1] Zizi-Sermpetzoglou A, Savvaidou V, Tepelenis N, Galariotis N, Olympitis M, Stamatiou K (2010). Sarcomatoid carcinoma of the prostate: a case report. *International Journal of Clinical and Experimental Pathology*.

[2] Eble JN, Sauter G, Ebstein JI, Sesterhenn IA (2004). *Pathology and Genetics of Tumors of the Urinary System and Male Genital Organs*.

[3] Grignon DJ (2004). Unusual subtypes of prostate cancer. *Modern Pathology*.

[4] Humphray P (2012). Histological variants of prostatic carcinoma and their significance. *Histopathology*.

[5] Hansel DE, Epstein JI (2006). Sarcomatoid carcinoma of the prostate: a study of 42 cases. *The American Journal of Surgical Pathology*.

[6] Zhou M, Netto GJ, Ebstein JI (2012). *Uropathology*.

